# Identification of Black Cumin (*Nigella sativa*) MicroRNAs by Next-Generation Sequencing and Their Implications in Secondary Metabolite Biosynthesis

**DOI:** 10.3390/plants13192806

**Published:** 2024-10-08

**Authors:** Andrea G. Uriostegui-Pena, Almendra Reyes-Calderón, Claudia Gutiérrez-García, Aashish Srivastava, Ashutosh Sharma, Sujay Paul

**Affiliations:** 1 NatProLab, School of Engineering and Sciences, Tecnologico de Monterrey, Queretaro 76130, Mexico; 2 Department of Clinical Science, University of Bergen, 5021 Bergen, Norway

**Keywords:** microRNA (miRNA), medicinal plants, secondary metabolism, high-throughput sequencing, black cumin/black seed

## Abstract

Secondary metabolites are bioactive compounds believed to contribute to the pharmacological properties of plants. MicroRNAs (miRNAs) are small non-coding RNA molecules involved in post-transcriptional regulation and are thought to play an important role in regulating secondary metabolism biosynthesis. Nevertheless, the extent of miRNA involvement in secondary metabolism remains minimal. *Nigella sativa* (black cumin/black seed) is a popular medicinal and culinary plant known for its pharmaceutical properties; however, its genomic information is scarce. In this study, next-generation sequencing (NGS) technology was employed to obtain the miRNA profile of *N. sativa*, and their involvement in secondary metabolite biosynthesis was explored. A total of 25,139,003 unique reads ranging from 16 to 40 nucleotides were attained, out of which 240 conserved and 34 novel miRNAs were identified. Moreover, 6083 potential target genes were recognized in this study. Several conserved and novel black cumin miRNAs were found to target enzymes involved in the terpenoid, diterpenoid, phenylpropanoid, carotenoid, flavonoid, steroid, and ubiquinone biosynthetic pathways, among others, for example, beta-carotene 3-hydroxylase, gibberellin 3 beta-dioxygenase, trimethyltridecatetraene synthase, carboxylic ester hydrolases, acetyl-CoA C-acetyltransferase, isoprene synthase, peroxidase, shikimate O-hydroxycinnamoyltransferase, etc. Furthermore, sequencing data were validated through qPCR by checking the relative expression of eleven randomly selected conserved and novel miRNAs (nsa-miR164d, nsa-miR166a, nsa-miR167b, nsa-miR171a, nsa-miR390b, nsa-miR396, nsa-miR159a, nsa-miRN1, nsa-miRN29, nsa-miRN32, and nsa-miRN34) and their expression patterns were found to be corroborated with the sequencing data. We anticipate that this work will assist in clarifying the implications of miRNAs in plant secondary metabolism and aid in the generation of artificial miRNA-based strategies to overproduce highly valuable secondary metabolites from *N. sativa*.

## 1. Introduction

*Nigella sativa* L. (Ranunculaceae), commonly known as black cumin or black seed, is an annual herbaceous flowering medicinal plant native to the Eastern Mediterranean, North Africa, the Indian subcontinent, and Southwest Asia [1,2]. It is mainly cultivated in Mediterranean countries, central Europe, western Asia, and the Middle East, including countries such as India, Egypt, Albania, Greece, Syria, Turkey, Saudi Arabia, and Pakistan [2,3,4]. *N. sativa* seeds are 1–5 mm in size and are commonly used as a spice and flavoring agent for bread, cheese, yogurt, pickles, and sauces [1,5]. Black cumin has also been used as a herbal medicine due to its antioxidant, analgesic, anti-inflammatory, immunomodulatory, anticancer, neuroprotective, antimicrobial (antibacterial, antifungal, antiviral, and antiparasitic), antihypertensive, cardioprotective, antidiabetic, spasmolytic, bronchodilator, gastroprotective, hypolipidemic, nephroprotective, and hepatoprotective properties [1,2,3,4,5,6]. *N. sativa* contains a variety of biochemical components, including quinones (thymoquinone, dithymoquinone, thymohydroquinone), terpenes (*α*-phellandrene, carvacrol, p-cymene, *α*-pinene, 4-terpineol, thymol), phenols, and flavonoids, as well as oleic acid, proteins, and carbohydrates [7,8]. Its major active chemical constituent, thymoquinone (TQ), has substantial antioxidant, anti-proliferative, antimicrobial, anti-analgesic, anticancer, anti-inflammatory, antineoplastic, antimutagenic, antiulcer, hypoglycemic, and wound healing properties, and also promotes immunity, cell survival, and energy metabolism [1,2,6,7,8].

MicroRNAs (miRNAs) are short endogenous single-stranded non-coding RNA molecules that regulate gene expression post-transcriptionally by either translational inhibition or mRNA degradation [9,10,11]. In plants, miRNAs are transcribed by the DNA-dependent RNA Polymerase II (Pol II) into a primary transcript (pri-miRNA) with a 5′-cap and a polyadenylated tail [12,13,14,15]. The imperfect stem–loop structure of this transcript is then recognized and cleaved by the Dicer-like RNase-II endonuclease 1 (DCL1) to form miRNA/miRNA * duplexes, which are later exported into the cytoplasm, where they combine with Argonaute (AGO) protein to form the RNA-induced silencing complexes (RISCs) that are involved in gene regulation [12,13,14,16]. miRNAs can be classified into conserved miRNAs that are usually highly expressed and evolutionary conserved among related plant genera, and non-conserved miRNAs that are species-specific, have low expression, or are induced under specific conditions [16,17,18]. These tiny molecules are involved in the regulation of developmental activities, including leaf morphogenesis and polarity, vegetative phase change, seed emergence, root morphology, flowering time and patterns, the regulation of meristem characteristics, and the response to biotic and abiotic stress, as well as in biological processes such as cell and tissue differentiation, proliferation, apoptosis, and metabolism [12,19,20,21,22]. Intriguingly, a number of plant miRNAs play a major role in controlling secondary metabolism, thus regulating phytochemical processes related to the plant’s interaction with the environment [20,23].

It has been shown that artificial miRNAs (amiRNAs) can be used to target mRNA transcripts to improve secondary metabolite production in planta. They are highly efficient and specific, with the ability to redirect endogenous miRNA biogenesis and silencing machinery in order to silence specific target RNAs [24]. They have been successfully applied for manipulating secondary metabolite biosynthesis [25]. For example, an amiRNA that targeted NtFLS expression was employed to study the effect on insect tolerance in *AtMYB12*-expressing tobacco. This amiRNA suppressed the increased production of rutin, a flavonoid with insecticidal effects induced by the overexpression of the transcription factor *AtMYB12* [26]. In a similar manner, the artificial miRNA target mimic (MIM858) was employed to demonstrate that miR858a targets flavanol-specific MYBs that are involved in the early regulation of the flavonoid biosynthetic pathway [27]. Nevertheless, a more thorough understanding of the mechanism by which amiRNAs work is still required to utilize them in the development of strategies to enhance the production of secondary metabolites.

Small-RNA sequencing is a type of next-generation sequencing (NGS) technology that can create a detailed map of an organism’s microRNA expression profile [28,29]. This study seeks to produce an NGS-based miRNA profile of *N. sativa*, a genetically poorly characterized species. Additionally, it examines the influence of miRNAs on biosynthetic pathways of secondary metabolism. The acquired information may facilitate the development of novel techniques, including amiRNAs, to regulate the production of secondary metabolites, which confer biomedical qualities to black cumin.

## 2. Results

### 2.1. Sequence Analysis of N. sativa Small RNAs

**Figure 1:**
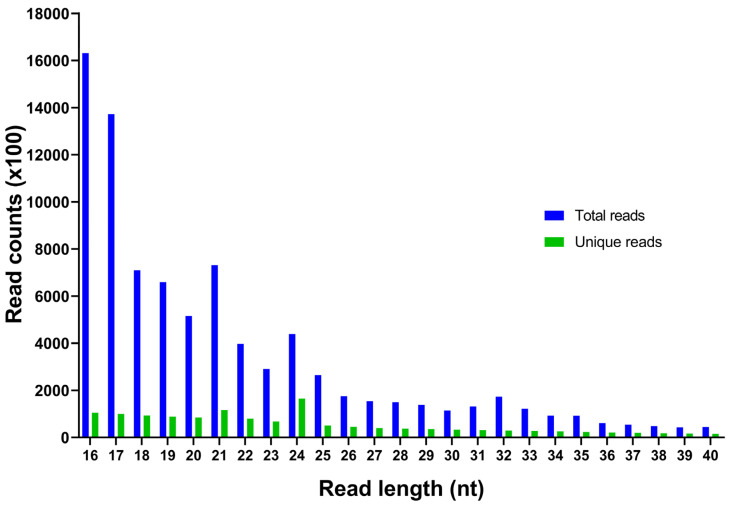
Size distribution and abundance of small RNA sequences identified in *Nigella sativa* through Illumina sequencing.

In this study, a total of 33,508,811 raw reads from *N. sativa* seedling samples were obtained through Illumina sequencing (NCBI SRA accession number: SRX25016763). After the removal of the adaptors, low-quality reads, and small RNAs, including rRNA (687,484), snoRNA (30,086), snRNA (20,189), and tRNA (4833), 25,189,003 unique reads with lengths between 16 and 40 nt were acquired, out of which 240 known and 34 novel miRNAs were identified (Table 1). The size distribution of unique *N. sativa* reads demonstrated that 24 nt were the most frequent (12.05%), followed by 21 nt (8.51%), 16 nt (7.67%), and 17 nt (7.30%) (Figure 1). The two major types of small RNAs include miRNAs and heterochromatic small interference RNAs (hc-siRNAs), the latter ones usually being 24 nt in length.

### 2.2. Identification of Conserved and Novel miRNAs in N. sativa

**Figure 2:**
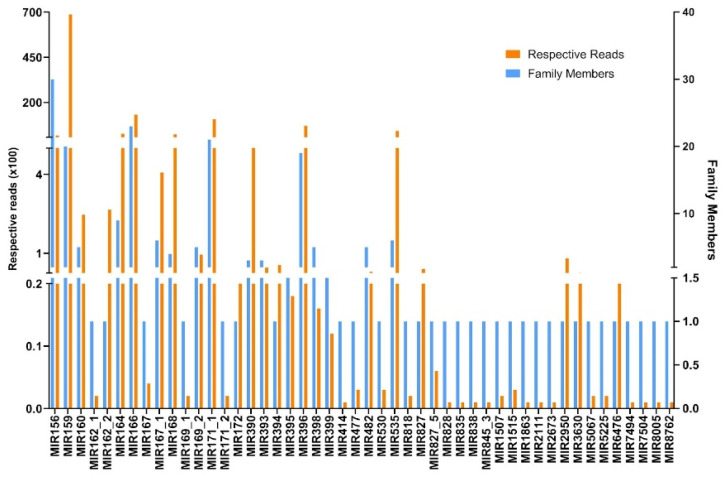
miRNA families with their respective number of members found in *N. sativa*.

For the identification of conserved miRNAs in *N. sativa*, the obtained unique reads were matched against miRbase-22 (https://www.mirbase.org/) (accessed on 23 August 2023) with the BLASTn tool. A total of 240 conserved miRNAs from 48 miRNA families were identified (please refer to the Supplementary Material for more information). All conserved miRNAs presented significant homology with no more than one mismatch with their respective homologs, and their frequency between families varied greatly. The most abundant miRNA families were miR156, miR166, miR171_1, and miR159, with 30, 23, 21, and 20 members, respectively (Figure 2). The read counts spanned from 1 to 68,731, where the miR159 family had the highest number of reads (68,731), followed by the miR166 (13,117), miR171_1 (10,556), and miR396 (6958) families.

**Figure 3:**
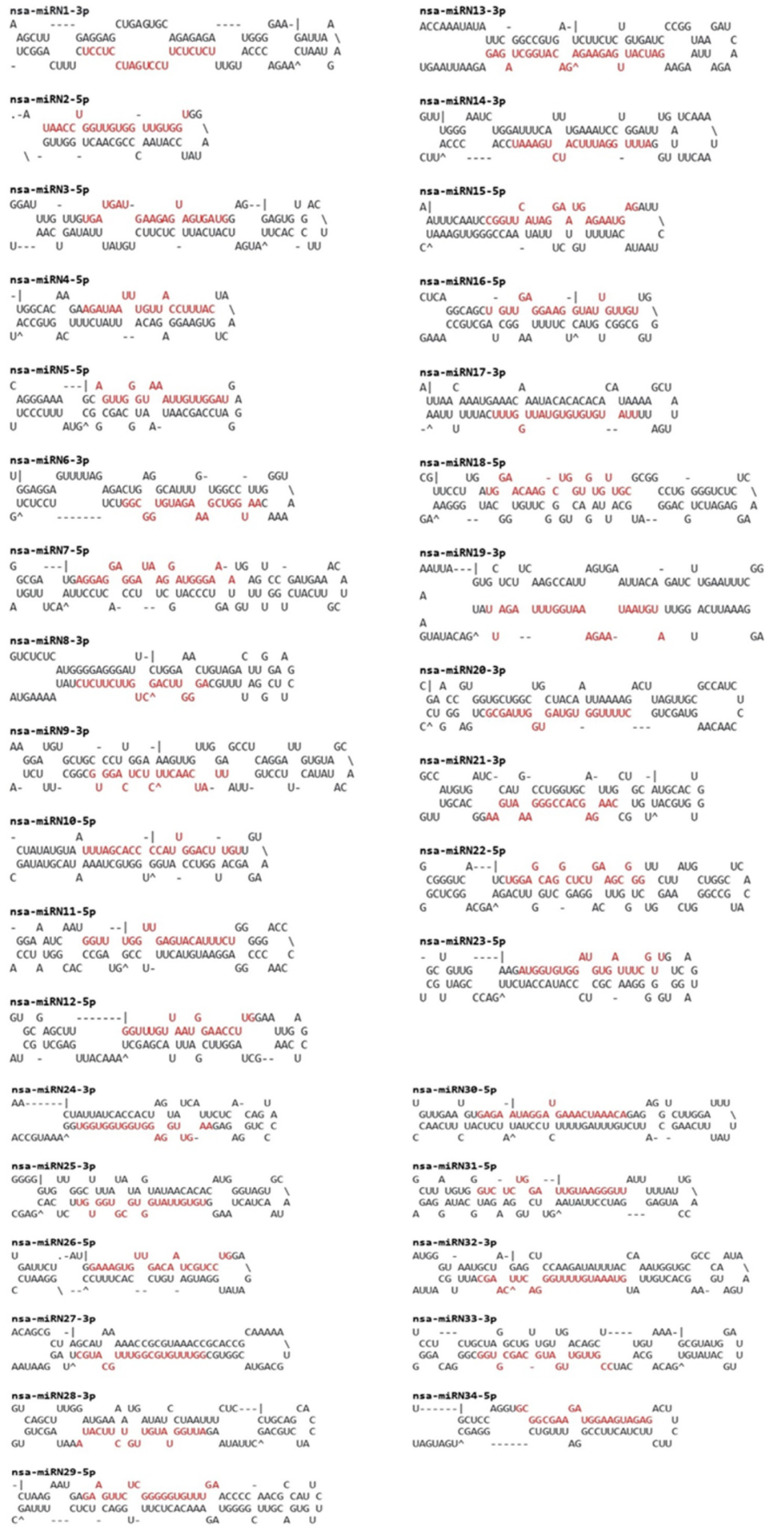
Secondary structures (stem–loops) of *N. sativa* novel miRNA precursors. Mature miRNAs are highlighted in red.

After the identification of conserved miRNAs, the remaining unaligned reads were subjected to predict novel *N. sativa* miRNA candidates by applying strict filtering criteria. A total of 34 novel black cumin miRNAs were identified in this study (Table 2), and their secondary hairpin structures were predicted (Figure 3). The read counts of these novel miRNA candidates ranged from one to three. The highest number of reads (three) were displayed by nsa-miRN1-3p, nsa-miRN24-3p, nsa-miRN29-5p, and nsa-miRN34-5p. The precursor sequences of these novel miRNA candidates had high minimum folding free energy index (MFEI) values, ranging from 0.71 to 1.53, with an average of 0.87 ± 0.21, differentiating them from other types of RNA, including tRNAs (0.64), rRNAs (0.59), and mRNAs (0.65) [30].

### 2.3. Target Prediction of Conserved and Novel N. sativa miRNAs and Their Functional Analysis

**Figure 4:**
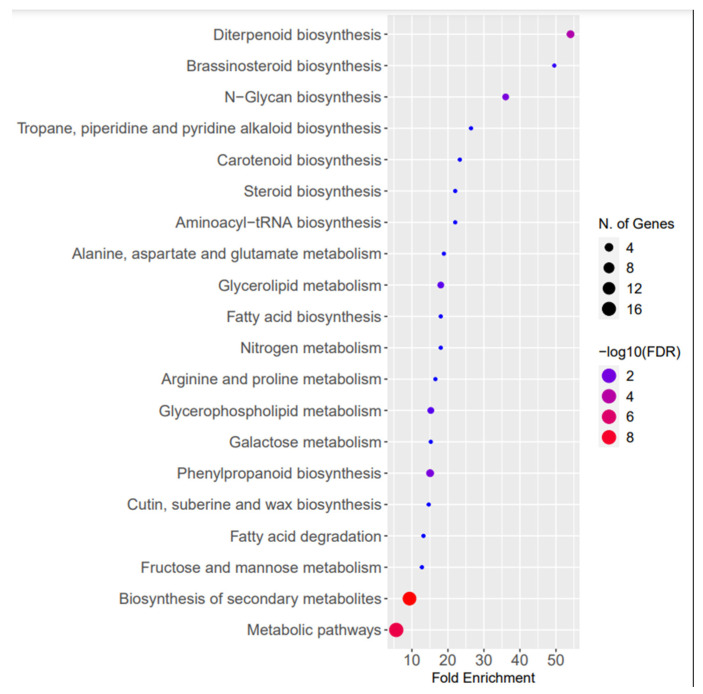
Pathway enrichment analysis of the potential target genes of *N. sativa* miRNAs.

In this study, a total of 6083 corresponding potential target genes of *N. sativa* miRNAs were identified (3071 target genes for conserved miRNAs and 3012 for novel miRNAs). The conserved miRNAs with the highest number of target genes included nsa-miR156e (295), nsa-miR164b-5p (137), and nsa-miR156j (123), while the novel ones included nsa-m0001-3p (216), nsa-m0038-3p (191), and nsa-m0036-5p (159). The pathway enrichment analysis revealed a greater number of genes associated with “Metabolic pathways” and “Biosynthesis of secondary metabolites”, followed by “Phenylpropanoid biosynthesis” and “Diterpenoid biosynthesis”. Additionally, the FDR values for “Metabolic pathways” and “Biosynthesis of secondary metabolites” were found to be the highest. The fold enrichment value represents the percentage of genes belonging to the respective pathways in comparison to the background frequency [31]. The highest fold enrichment value was obtained by “Diterpenoid biosynthesis”, followed by “Brassinosteroid biosynthesis” (Figure 4).

### 2.4. Identification of N. sativa miRNA Targets Involved in Plant Secondary Metabolite Biosynthesis

The secondary metabolites produced by different enzymes present in *N. sativa* are thought to be responsible for its medicinal properties. Thus, the aim of this study was to identify the *N. sativa* miRNAs that target genes that code for enzymes involved in secondary metabolism biosynthesis pathways. The obtained results revealed several *N. sativa* target genes associated with carotenoid, diterpenoid, phenylpropanoid, and the terpenoid backbone, among other various plant secondary metabolites (Table 3). For example, two miRNAs (nsa-miR167c and nsa-miR167b) indicated control over the enzyme 15-cis-phytoene synthase, while nsa-miR157d-3p regulates beta-carotene 3-hydroxylase, both involved in carotenoid biosynthesis. Similarly, four miRNAs (nsa-miR394b-5p, nsa-miR159, nsa-miR535d, and nsa-miR169d) were found to be involved in diterpenoid synthesis through targeting a variety of enzymes (gibberellin-44 dioxygenase, gibberellin 2beta-dioxygenase, gibberellin 3beta-dioxygenase, and trimethyltridecatetraene synthase). Three enzymes involved in the phenylpropanoid biosynthesis pathways were found to be targeted by different *N. sativa* miRNAs as well; carboxylic ester hydrolases were targeted by nsa-miR8175, nsa-miR156r, nsa-miR156f, nsa-miR156w, nsa-miR156b, nsa-miR156p, and nsa-miR156l; coniferyl aldehyde dehydrogenase by nsa-miR164b-5p; and peroxidase by nsa-miR156j, nsa-miR156e, nsa-miR156q, nsa-miR164c, nsa-miR164c-5p, and nsa-miR164b-5p. In the biosynthesis of various secondary metabolites, the nsa-miR399i targeted 3″-deamino-3″-oxonicotianamine reductase for the biosynthesis of mugineic acid, while nsa-miR167c and nsa-miR167b targeted the enzyme scopoletin glucosyltransferase during the phenylpropanoid biosynthesis. Lastly, terpenoid backbone biosynthesis enzymes, including acetyl-CoA C-acetyltransferase, 1-deoxy-D-xylulose-5-phosphate synthase, 2-C-methyl-D-erythritol 4-phosphate cytidylyltransferase, (E)-4-hydroxy-3-methylbut-2-enyl-diphosphate synthase, isoprene synthase, and CAAX prenyl protease 2, were targeted by nsa-miR156w, nsa-miR535d, nsa-miR157d-3p, nsa-miR156e, nsa-miR171a-3p, and nsa-miR156j, respectively.

Interestingly, several targets of *N. sativa* novel miRNAs were also found to be involved in secondary metabolism biosynthesis pathways, including the terpenoid backbone, flavonoid, steroid, ubiquinone, and other terpenoid–quinone biosynthesis pathways (Table 4). The miRN1-3p was linked to the enzyme shikimate O-hydroxycinnamoyltransferase in both the flavonoid and phenylpropanoid biosynthesis pathways, as well as to two enzymes (acetyl-CoA C-acetyltransferase and 2-C-methyl-D-erythritol 4-phosphate cytidylyltransferase) in the terpenoid backbone biosynthesis pathway. In a similar manner, nsa-mRN24-3p was revealed to target the enzymes shikimate O-hydroxycinnamoyltransferase in flavonoid and phenylpropanoid biosynthesis, peroxidase in phenylpropanoid biosynthesis, and sterol 24-C-methyltransferase and plant 3beta-hydroxysteroid-4alpha-carboxylate 3-dehydrogenase in the steroid biosynthesis pathway. In the biosynthesis of phenylpropanoid, peroxidase was also targeted by nsa-mRN10-5p; this miRNA was found to be correlated to isopentenyl phosphate kinase in the terpenoid backbone biosynthesis pathway as well. Finally, the enzymes homogentisate phytyltransferase and homogentisate geranylgeranyltransferase were targeted by nsa-miRN16-5p within the ubiquinone and other terpenoid–quinone biosynthesis pathways.

### 2.5. Experimental Validation of N. sativa miRNAs by qPCR

**Figure 5:**
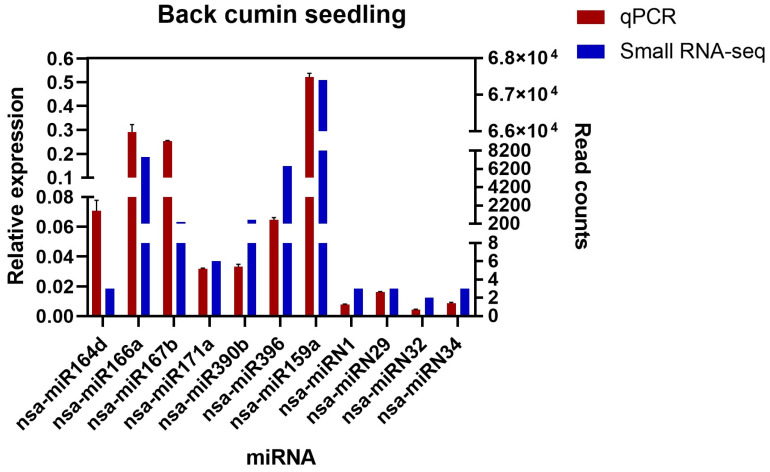
Quantitative PCR analysis of novel and conserved miRNAs from *N. sativa*. The relative expression of seven conserved miRNAs (nsa-miR164d, nsa-miR166a, nsa-miR167b, nsa-miR171a, nsa-miR390b, nsa-miR396, and nsa-miR159a) and four novel miRNAs (nsa-miRN1, nsa-miRN29, nsa-miRN32, and nsa-miRN34) was detected by qPCR. U6 was used as an endogenous control, and the analysis was performed in triplicate.

In this study, seven conserved and four novel *N. sativa* miRNAs were randomly selected for qPCR analysis to validate the data obtained by high-throughput sequencing. A similar relative expression of all analyzed miRNAs was observed with respect to the read counts (Figure 5).

## 3. Discussion

As an adaptive response to biotic and abiotic stresses, plants produce secondary metabolites (SMs) to ensure functional flexibility without altering their cellular and developmental physiological processes [31,32]. SMs are of great importance due to their applications in the cosmetic, antifeedant, pigment, and pharmaceutical industries [33]. miRNAs are small, endogenous, non-coding RNA molecules capable of regulating gene expression post-transcriptionally and plant secondary metabolism biosynthesis due to their involvement in the transduction of the peripheral metabolic series [34,35]. Thus, SMs can be artificially regulated by miRNAs to enhance their production [35]. In this study, Illumina small-RNA sequencing was performed to establish the miRNA profile of *N. sativa* with the objective of better understanding their involvement in plant secondary metabolism.

Results showed that the conserved miRNAs from young seedlings of *Nigella sativa* were distributed among 64 miRNA families, with the miR156 family having the highest number of family members (30), while the miR159 family had the maximum read count (68,731). It is well established that miR156 expression is highly conserved in plants, and it is associated with the development of juvenile plants and with the delay of phase transition, which therefore concurs with its presence in the young seedlings of black cumin that were studied [36]. The miR156 family was also found to be one of the families with the greatest number of members in *Mentha* spp. and is thought to be involved in the regulation of essential oil biosynthesis due to its modulation of the terpenoid backbone synthesis pathways [37]. Moreover, miR156 targets squamosa promoter-binding protein-like (SPL) factors that play an important role in the regulation of sesquiterpene biosynthesis, including (E)-β-caryophyllene in *Arabidopsis thaliana* [38]. On the other hand, miR159a is supposed to be involved in phenolic acid biosynthesis in *S. miltiorrhiza* [39].

In our study, 24 nt is the predominant size in unique *N. sativa* reads. While miRNAs can range from 21 to 24 nt, other small non-coding RNAs, including heterochromatic siRNAs (hc-siRNAs), are primarily 24 nt in length. They originate from repetitive regions or transposons and contribute to the preservation of genomic integrity by promoting heterochromatin formation [40,41]. Both miRNAs and hc-siRNAs induce silencing via base-pairing interactions with target RNAs. However, unlike miRNAs, which are derived from single-stranded primary RNAs, hc-siRNAs are processed from double-stranded RNA and regulate silencing through RNA-directed DNA methylation [42]. A distinct secondary structure of the precursor and some strict filtering criteria can differentiate between miRNA and hc-siRNA [18].

Although the phytochemical composition of black cumin varies depending on factors such as maturity state, place of growth, processing methods, and isolation techniques, phytochemicals, including terpenes and terpenoids, flavonoids, phytosterols, alkaloids, and polyphenols, have been identified to be present in *N. sativa* [2,43,44]. These bioactive constituents are considered to contribute to the biomedical activities of this plant [45]. The most common bioactive component of *N. sativa* is TQ, followed by thymohydroquinone (THQ), dithymoquinone, p-cymene, carvacrol, t-anethole, and α-pinene, among others [46,47]. TQ is a monoterpene benzoquinone synthesized from γ-terpinene during secondary metabolism that confers its antioxidant, anti-neoplastic, anti-inflammatory, and immunomodulatory effects [2,45,48]. Current results showed that key enzymes involved in the terpenoid backbone, diterpenoid, carotenoid, phenylpropanoid, steroid, ubiquinone and other terpenoid–quinone biosynthesis pathways are regulated by both novel and conserved miRNAs.

Carotenoids are pigments that function as sources for apocarotenoid metabolites, which trigger adaptive responses to stress conditions, including high light, high salt, and drought [49]. The carotenoid biosynthesis starts with the generation of 15-*cis*-phytoene, involved in the regulation of the flow of carotenoids [50] and which was found to be targeted by two conserved miRNAs (nsa-miR167c and nsa-miR167b) in the carotenoid biosynthesis pathway in *N. sativa*. Additionally, conserved nsa-miR157d-3p was found to control beta-carotene 3-hydroxylase, an important enzyme necessary for zeaxanthin biosynthesis, which is a carotenoid known for its antioxidant and anti-inflammatory properties [51,52,53].

Terpenoids are the largest group of plant secondary metabolites that are involved in the interactions and defense reactions in plants [54]. There are two mechanisms by which the universal precursors of terpenoids, dimethylallyl diphosphate (DMAPP) and isopentenyl diphosphate (IPP), can be obtained, though the mevalonate (MVA) and the methyl erythritol phosphate (MEP) pathways [55]. Generally, the MEP pathway provides C_5_ prenyl diphosphates for C_10_ monoterpene, C_20_ diterpene, and C_40_ tetraterpene biosynthesis, while the MVA supplies the same universal precursors for the biosynthesis of C_15_ sesquiterpenes, C_27–29_ sterols, C_30_ triterpenes, and their saponin derivatives [56]. Acetyl-CoA C-acetyltransferase (AACT) is a regulatory enzyme of terpenoid biosynthesis that is responsible for the condensation of two acetyl-CoA to form acetoacetyl-CoA at the beginning of the MVA biosynthesis pathway, and it promotes squalene production under salt stress [57,58]. AACT was found to be targeted in terpenoid backbone biosynthesis by conserved nsa-miR156w and novel nsa-mRN1-3p in *N. sativa*. Corroborating our results, six miRNAs associated with SM biosynthesis were also identified in the medicinal plant *Swertia chirayita*, including miR-168, miR-11320, miR-166a, miR-11071, miR-156a, and miR-166b, found to target AACT, aspartate aminotransferase (PHAT), premnaspirodiene oxygenase (PSO), ribulose-phosphate 3-epimerase (RPE), phosphoglycerate mutase (PGM), and a gene-encoding homeobox-leucine zipper protein (HD-ZIP) [59]. 1-deoxy-D-xylulose-5-phosphate synthase (DXS) is the first and rate-limiting enzyme to produce IPP and DMAPP as terpenoid biosynthesis precursors via the MEP pathway [60], and was observed to be targeted by nsa-miR535d in this study. Moreover, the novel nsa-miRN10-5p was identified to target isopentenyl phosphate kinase (IPK), an enzyme that increases terpenoid production through the MVA and MEP pathways and responsible for the transformation of isopentenyl phosphate (IP) and dimethylallyl phosphate (DMAP) to IPP and DMAPP through ATP-dependent phosphorylation [61]. 2-C-methyl-D-erythritol 4-phosphate cytidylyltransferase (IspD) is an enzyme involved in the mevalonate-independent pathway of terpenoid biosynthesis. It catalyzes 2-C-Methyl-D-erythritol 4-phosphate (MEP) and cytosine triphosphate (CTP) to 4-diphosphocytidyl-2-C-methyl-D-erythritol (CDPME) and inorganic pyrophosphate (PPi) [62]. Both conserved (nsa-miR157d-3p) and novel (nsa-mRN1-3p) miRNAs were identified to target this enzyme in *N. sativa*. Likewise, nsa-miR156e targeted (E)-4-hydroxy-3-methylbut-2-enyl-diphosphate synthase (GcpE), which takes part in the catalysis of the conversion of 2-C-methyl-d-erythritol cyclodiphosphate (MEcPP) into (E)-4-hydroxy-3-methylbut-2-enyl diphosphate (HMBPP) for terpenoid biosynthesis through the MEP pathway [63]. Lastly, isoprene synthase was found to be regulated by nsa-miR171a-3p. This enzyme catalyzes the conversion of DMAPP into isoprene in the MEP pathway and is produced to protect plants from abiotic stress [64].

Gibberellins (GAs) are involved in plant development and stress response signaling, and their bioactive levels are regulated by gibberellin–dioxygenases [65,66]. In this study, the enzymes gibberellin-44 dioxygenase, gibberellin 2beta-dioxygenase, and gibberellin 3beta-dioxygenase were targeted by conserved miRNAs such as nsa-miR394b-5p, nsa-miR159, and nsa-miR535d, respectively, in the diterpenoid biosynthesis pathway. In this pathway, nsa-miR169d targeted trimethyltridecatetraene synthase, also known as CYP82G1, an enzyme involved in the final step of the biosynthesis of C16-homoterpene (E,E)-4,8,12-trimethyltrideca-1,3,7,11-tetraene (TMTT) and C11-homoterpene (E)-4,8-dimethyl-1,3,7-nonatriene (DMNT), two homoterpene volatiles that are produced in response to herbivore attacks [67].

Plant peroxidases (PODs) are ubiquitous enzymes involved in the catalysis of the oxidation of substances at the expense of hydrogen peroxide (H_2_O_2_) [68]. An increase in POD concentration is associated with a plant’s response to stress and with a greater antioxidant status due to the stimulation of phenylpropanoid biosynthesis under stress, thus producing compounds with antioxidant potential [69,70]. Six conserved (nsa-miR156j, nsa-miR156e, nsa-miR156q, nsa-miR164c, nsa-miR164c-5p, and nsa-miR164b-5p) and three novel (nsa-mRN1-3p, nsa-mRN10-5p, and nsa-mRN24-3p) miRNAs were found to target POD in the phenylpropanoid biosynthesis pathway in *N. sativa*. In this pathway, other enzymes such as carboxylic ester hydrolases (CEHs) and coniferyl aldehyde dehydrogenase (CALDH) were also identified to be targeted by seven (nsa-miR8175, nsa-miR156r, nsa-miR156f, nsa-miR156w, nsa-miR156b, nsa-miR156p, and nsa-miR156l) and one (nsa-miR164b-5p) conserved miRNAs, respectively. CEHs are ubiquitous enzymes that catalyze the hydrolysis of ester bonds into alcohols and carboxylic acids [71], while CALDH is involved in the oxidation of coniferyl aldehyde to ferulic acid, which is a metabolite of the phenylpropanoid pathway that has a role in cell wall structure and recalcitrance [72].

Methyltransferases (MTases) were found to be targeted by novel miRNAs in the flavonoid and steroid biosynthesis pathways. MTases can transform metabolites involved in defense response, pigments, and cell signaling in plants and are categorized depending on the methyl-accepting atom (O, N, C, S, or Se) [73]. O-methylation plays an important role in stress tolerance and disease resistance in plants [74], and current results showed that the enzyme shikimate O-hydroxycinnamoyltransferase was targeted by novel nsa-mRN1-3p and nsa-mRN24-3p in flavonoid and phenylpropanoid biosynthesis. In a similar manner, the enzyme sterol 24-C-methyltransferase (SMT1 and ERG6) was targeted by nsa-mRN24-3p in steroid biosynthesis. Plant 3beta-hydroxysteroid-4alpha-carboxylate 3-dehydrogenase, also known as 3beta-hydroxysteroid dehydrogenases/C-4 decarboxylase (3βHSD/D), was also found to be targeted by nsa-miRN24-3p in steroid biosynthesis. This enzyme is responsible for the NAD^+^-dependent catalysis of the oxidative decarboxylation of 4α-carboxysterol intermediates that are involved in the C-4 demethylation of sterol precursors [75].

In the ubiquinone and other terpenoid–quinone biosynthesis pathways, nsa-miRN16-5p was linked to the enzymes homogentisate phytotransferase (HPT) and geranylgeranyl transferase (HGGT), respectively. They are critical enzymes that catalyze the condensation of homogentisate and C_20_ isoprenoid phytyl diphosphate (PDP) and geranylgeranyl diphosphate to form 2-methyl-6- phytylbenzoquinone, involved in tocopherol (vitamin E) biosynthesis as a mechanism of response of plants to adapt to various stress conditions [76]. Lastly, other conserved miRNAs were found to target enzymes involved in the biosynthesis of various secondary metabolites. For instance, nsa-miR399i appeared to target 3″-deamino-3″-oxonicotianamine, an enzyme involved in mugineic acid biosynthesis, while nsa-miR167c and nsa-miR167b targeted scopoletin glucosyltransferase, which converts scopoletin into scopolin, which are coumarin derivatives linked to reactive oxygen species (ROS) scavenging and pathogen defense [77].

Recently, similar studies have been performed to establish different medicinal plants’ miRNA profiles to understand their involvement in secondary metabolism. For example, Gutiérrez-García and colleagues (2022) studied curry (*Murraya koenigii*) leaves and identified miRNAs implicated in terpenoid and flavonoid biosynthesis, including novel miRNAs such as mko-miR5082, mko-miR396g-5p, mko-miRN7-3p, mko-miR827b, mko-miR167a, mko-mir156, mko-mir396, and mko-miRN4-5p, as well as conserved miRNAs such as miRNAs mko-miR168b, mko-miR858, mko-miR508, and mko-miR8610.1 [78]. In a similar manner, Reyes-Calderón and collaborators (2023) identified miRNAs in the seedlings of cumin (*Cuminum cyminum*), such as cci-miR156q, cci-miR157b-3p, cci-miR159, cci-miR159c, cci-miR166d-5p, cci-miR172c-5p, cci-miR172d-5p, cci-miR319d-3p, cci-miR394b-5p, cci-miR396a-3p, cci-miR397, cci-miR2118, cci-miR5564b, cci-miR6476a, cci-miRN3-5p, cci-miRN19-3p, cci-miRN32-5p, and cci-miRN34-3p, to be involved in the terpenoid backbone biosynthesis pathway, as well as the novel cci-miRN2-3p implicated in the carotenoid biosynthesis pathway [16]. Kaur et al. (2023) found in leaf and root tissues from Kuth (*Saussurea lappa*) conserved miRNAs such as miR171c.1, miR6106, miR164a.1, miR171b.1, and miR405a, as well as novel miRNAs including sla-miR121, sla-miR624, sla-miR765, and sla-miR897 to be involved in secondary metabolite biosynthesis. Out of these miRNAs, miR171c.1 and sla-miR121 were found to be implicated in the sesquiterpenoid biosynthesis pathway [79]. The response of wheat cultivars to salinity stress was examined through their miRNA differentiation expression profile. Findings revealed that tae-miR156, tae-miR160, tae-miR171a-b, tae-miR319, tae-miR159a-b, tae-miR9657, and novel-mir59 play a role in the modulation of salt tolerance by regulating ARF, SPL, SCL6, PCF5, R2R3 MYB, and CLB-CIPK, which may be implicated in cell growth, ion homeostasis, hormone signaling, redox maintenance, and stress defense mechanisms [80].

Validation of the sequencing analysis revealed an analogous expression pattern between the Illumina sequencing and the qPCR assay. A similar pattern of the results was also observed in the roots of *Arabidopsis thaliana* in response to cold exposure [81] and on *Fagopyrum tataricum* seeds during their development [82].

## 4. Materials and Methods

### 4.1. Plant Materials, RNA Extraction, and Quality Control

*N. sativa* seeds were collected from a local supermarket and manually stirred for 15 min in distilled water with colloidal silver (10 drops of Microdacyn^®^ in 100 mL of water), followed by washing with 5% sodium hypochlorite (200 μl tween in 100 mL sodium hypochlorite) for 20 min. Subsequently, after the seeds had been washed with distilled water, they were manually stirred on fungicide for 10 min and further washed with distilled water. The process of seed germination involved placing the seed in flasks containing MS medium, which were then incubated at 25 °C for a period of 15 days with a 12 h light/dark photoperiod. Healthy *N. sativa* seedling samples were collected and instantly frozen in liquid nitrogen. Total RNA (including small RNA) was extracted from frozen seedling samples with the Direct-zol RNA micro Kit (Zymo Research, Irvine, CA, USA) following the manufacturer’s instructions. RNA concentration and purity were assessed using a Nanodrop spectrophotometer (Thermo Scientific, Waltham, MA, USA), the integrity of the samples was determined on Tapestation (Agilent, Santa Clara, CA, USA), and the concentration was quantified with the Qubit RNA hs assay kit (Thermo Scientific, Waltham, MA, USA).

### 4.2. Small-RNA Library Construction and Sequencing

A small-RNA sequencing library was constructed using the QIAseq^®^ miRNA Library Kit (Qiagen, Germantown, MD, USA) following the manufacturer’s protocol. Briefly, 63 ng of total RNA was used as commencing material, and 3′ adapters were ligated to the specific 3′ OH group of miRNAs, followed by the ligation of the 5′ adapter. Subsequently, adapter-ligated fragments were reverse transcribed with the Unique Molecular Index (UMI) assignment, and the cDNA was enriched and barcoded by PCR amplification. The obtained cDNA library was then quantified by a Qubit fluorometer (Thermo Fisher Scientific, Waltham, MA, USA), while the size distribution analysis was performed on the Agilent 2200 TapeStation system. Finally, high-throughput sequencing was performed on the Illumina NovaSeq 6000 for 150 cycles, as per the manufacturer’s instructions.

### 4.3. Small-RNA Sequencing Data Analysis

Following Illumina sequencing, the raw data were processed by sRNA workbench (V3.0_ALPHA) to remove 3′ adapters, low-quality reads (<q30), reads <16 bp and >40 bp, and reads matching to other ncRNAs including rRNA, tRNA, and snoRNAs (the low quality and contaminated reads were removed on the following criteria to obtain the final clean reads: ➢ elimination of low-quality reads (<q30) ➢ elimination of 3′ adapters ➢ elimination of reads <16 bp and >40 bp). The remaining small-RNA sequences were aligned against miRbase22 (http://www.mirbase.org, accessed on 26 February 2024) for the identification of conserved *N. sativa* miRNAs by a homology approach against Viridiplantae miRNAs with 0 mismatches, and bowtie-1.1.1 was used to align the reads against the Rfam (non-coding) database with 3 mismatches. The sequences that did not display homology were considered for novel miRNA prediction using bowtie (https://bowtie-bio.sourceforge.net/index.shtml, accessed on 26 February 2024) and Mireap_0.22b. In this study, novel miRNAs were only considered if they displayed both suitable precursor secondary structures and MFEI values of ≥0.70. Prediction of the precursor’s secondary structure was performed with UNAFold Web Server (http://www.unafold.org/, accessed on 26 February 2024), and the MFEI values were calculated with the following formula:

$$MFEI=\frac{\left(\frac{MFE}{length\;of\;RNA\;sequence}\right)\times 100}{\%\;GC\;content}$$

### 4.4. Prediction of N. sativa miRNA Targets, Their Functional Annotation, and Pathway Analysis

For the target prediction of the known and novel miRNAs, the miRanda tool [83] was utilized. The pathway analysis of the potential *N. sativa* miRNA targets was performed on the KAAS server using common Eudicots plants as reference organisms. Lastly, the analysis of miRNA targets associated with pathways for the biosynthesis of secondary metabolites was performed manually.

### 4.5. Extraction of Small RNA and Experimental Validation of N. sativa miRNAs by qPCR

For the validation of the identified conserved and novel miRNAs, small RNA was extracted from frozen *N. sativa* seedlings with the miRNeasy Mini Kit (Qiagen, Hilden, NRW, Hamburg, Germany) following the manufacturer’s instructions. The quality and quantity of the samples were assessed with the NanoDrop One UV-Vis microvolume spectrophotometer (Thermo Scientific, Waltham, MA, USA). The resulting small RNAs were polyadenylated and reverse transcribed with the Mir-X-miRNA First-Strand Synthesis kit (Takara Bio USA, Inc., San Jose, CA, USA). Lastly, qPCR was performed using the TB Green ^®^ Advantage ^®^ qPCR Premix (Takara Bio USA, Inc., San José, CA, USA) in a StepOne ^™^ Real-Time PCR System (Applied Biosystems, Waltham, MA, USA). The reactions were performed in a 48-well optical plate with the following conditions: initial polymerase activation step at 95 °C for 10 s, denaturation by 45 cycles at 95 °C for 5 s, and annealing and extension at 55 °C for 20 s. The amplification cycle was followed by a melting curve analysis ranging from 56 to 95 °C and temperature increments of 0.5 °C every 10 s. The reactions were performed with three biological and technical replicates for each sample, and the relative expression of the miRNAs was normalized using U6 as an endogenous control.

## 5. Conclusions

In this study, a total of 240 conserved and 34 novel miRNAs from *N. sativa* seedlings were identified through Illumina high-throughput sequencing technology. Among these, three conserved miRNAs (nsa-miR167c, nsa-miR167b, and nsa-miR157d-3p) were found to target important enzymes involved in the carotenoid biosynthesis pathway, four (nsa-miR394b-5p, nsa-miR159, nsa-miR535d, and nsa-miR169d) in the diterpenoid biosynthesis pathway, thirteen (nsa-miR8175, nsa-miR156r, nsa-miR156f, nsa-miR156w, nsa-miR156b, nsa-miR156p, nsa-miR156l, nsa-miR156j, nsa-miR156e, nsa-miR156q, nsa-miR164c, nsa-miR164c-5p, and nsa-miR164b-5p) in phenylpropanoid biosynthesis, six in the terpenoid backbone biosynthesis pathway (nsa-miR156w, nsa-miR535d, nsa-miR157d-3p, nsa-miR156e, nsa-miR171a-3p, and nsa-miR156j), and three (nsa-miR399i, nsa-miR167c, and nsa-miR167b) in the biosynthesis pathway of various plant secondary metabolites. Likewise, two novel miRNAs (nsa-mRN1-3p and nsa-miRN24-3p) were discovered to regulate an enzyme associated with flavonoid biosynthesis, three (nsa-mRN1-3p, nsa-mRN10-5p, and nsa-miRN24-3p) in the phenylpropanoid, two (nsa-mRN1-3p and nsa-mRN10-5p) in the terpenoid backbone, one (nsa-miRN24-3p) in the steroid, and one (nsa-miRN16-5p) in the ubiquinone and other terpenoid–quinone biosynthesis pathways. Finally, qPCR analysis was employed to experimentally validate eleven random miRNAs (nsa-miR164d, nsa-miR166a, nsa-miR167b, nsa-miR171a, nsa-miR390b, nsa-miR396, nsa-miR159a, nsa-miRN1, nsa-miRN29, nsa-miRN32, and nsa-miRN34). To the best of our knowledge, this is the first report of the miRNA profile of the culinary and medicinal plant *N. sativa* and its involvement in secondary metabolite biosynthesis. The findings from this study could reinforce the miRNA-mediated transgenic research to overproduce medicinally important plant secondary metabolites.

## Figures and Tables

**Table 1:** Categorization of sequencing reads (analysis performed using sRNA workbench, bowtie, Mireap_0.22b, and UNAFold bioinformatic tools).

Category	Total Reads	Unique Reads
Raw data generated after sequencing	33,508,811	25,139,003
Trimmed reads	8,605,403	1,368,483
Reads aligned to miRBase	291,381	196,114
Known miRNA Uniq	240	
Reads utilized for novel miRNA	2,286,411	393,335
Novel miRNA predicted	34	
Putative miRNAs	2,168,726	355,560
% reads aligned to ncRNA	54.26%	
Reads aligned to ncRNA (rRNA, snoRNA, snRNA, tRNA)	6,027,611	742,592

**Table 2:** Potential novel miRNA candidates identified from *N. sativa*.

Name	Sequence (5′-3′)	Length	Read Count	Strand	MFEI of Precursor	Potential Targets Related to Secondary Metabolite Biosynthesis
nsa-miRN1-3p	UCUCUCUUCCUGAUCCUCCU	20	3	+	0.7317	7-deoxyloganetic acid glucosyltransferase-like acetyl-CoA acetyltransferase, cytosolic 1-like isochorismate synthase, chloroplastic-like
nsa-miRN2-5p	UAACCUGGUUGUGGUUGUGGU	21	2	−	0.7723	beta-D-glucosyl crocetin beta-1,6-glucosyltransferase-like UDP-glucosyltransferase 29-like
nsa-miRN3-5p	UGAUGAUGAAGAGUAGUGAUG	21	1	−	0.8129	probable methionine–tRNA ligase
nsa-miRN4-5p	AGAUAAUUUGUUACCUUUAC	20	2	−	1.2435	
nsa-miRN5-5p	AGUUGGGUAAAUUGUUGGAU	20	1	−	0.9194	NA
nsa-miRN6-3p	AAUGGUCGAAAGAUGUGGCGG	21	2	−	0.7568	NA
nsa-miRN7-5p	AGGAGGAUAAGGAUGGGAAA	20	2	−	0.7930	GPI transamidase component PIG-S-like
nsa-miRN8-3p	AGGGUUCAGCUGUUCUUCUC	20	2	+	0.7974	triacylglycerol lipase SDP1
nsa-miRN9-3p	UUAUCAACUUCUCUCAGGUG	20	1	+	0.7372	adenylate isopentenyltransferase 3, chloroplastic
nsa-miRN10-5p	UUUAGCACCCCAUUGGACUUGU	22	1	+	1.2967	peroxidase N1-like phosphatidylserine decarboxylase proenzyme 2 caffeic acid 3-O-methyltransferase
nsa-miRN11-5p	GGUUUGGUUGAGUACAUUUCU	21	1	+	0.7452	NA
nsa-miRN12-5p	GGUUUGUUAAUGGAACCUUG	20	1	−	0.7200	NA
nsa-miRN13-3p	GAGAAGAGACAUGGCUAGAG	20	2	−	0.7730	glutamine synthetase cytosolic isozyme long-chain acyl-CoA synthetase 6, peroxisomal palmitoyl-acyl carrier protein thioesterase, chloroplastic-like
nsa-miRN14-3p	AUUUGGAUUUCAUCUGAAAU	20	2	−	1.0207	NA
nsa-miRN15-5p	CGGUUCAUAGGAAUGAGAAUGAG	23	1	+	1.1087	beta-glucosidase BoGH3B-like 3-ketoacyl-CoA synthase 4
nsa-miRN16-5p	UGUUGAGGAAGGUAUUGUUGU	21	1	+	0.7914	NADPH/quinone oxidoreductase homogentisate phytyltransferase 1, chloroplastic
nsa-miRN17-3p	UUUAUGUGUGUGUAUUGGUUU	21	1	−	1.5333	NA
nsa-miRN18-5p	UGGAACAAGCUGGUGUGUUGCG	22	1	−	0.7264	NA
nsa-miRN19-3p	AUGUAAUAAGAAAUGGUUUAGAUU	24	2	+	0.8467	NA
nsa-miRN20-3p	CUUUUGGUGUAGUGGUUAGCG	21	1	−	0.7979	NA
nsa-miRN21-3p	CAAGAGCACCGGGAAAUGAA	20	1	+	0.7156	UDP-glycosyltransferase 85A8
nsa-miRN22-5p	UGGAGCAGGCUCUGAAGCGG	20	1	−	0.7328	gibberellin 20 oxidase 1-like biotin carboxyl carrier protein of acetyl-CoA carboxylase 1, chloroplastic-like CTP synthase-like
nsa-miRN23-5p	AUGGUGUGGAUGUGAUUUCGUU	22	1	+	0.7200	geranylgeranyl pyrophosphate synthase 7, chloroplastic-like
nsa-miRN24-3p	AAGUUGGAGGUGGUGGUGGU	20	3	−	0.8147	polyphenol oxidase I, chloroplastic bifunctional riboflavin biosynthesis protein RIBA 1, chloroplastic bifunctional riboflavin biosynthesis protein RIBA 1, chloroplastic
nsa-miRN25-3p	UGUGUUAUGGUGCGUGGUGU	20	1	−	0.7462	3-ketoacyl-CoA synthase 6-like heterodimeric geranylgeranyl pyrophosphate synthase small subunit, chloroplastic-like
nsa-miRN26-5p	GAAAGUGUUGACAAUCGUCCUG	22	1	−	0.7143	NA
nsa-miRN27-3p	GGUUUGUGCGGUUUGCAUGC	20	1	−	0.9659	S-adenosylmethionine decarboxylase proenzyme-like
nsa-miRN28-3p	AUUGGUAUGUUGUCUUCAUA	20	1	−	0.7543	glutamyl-tRNA(Gln) amidotransferase subunit A, chloroplastic/mitocondrial
nsa-miRN29-5p	GAAGUUCUCGGGGGUGUUUGA	21	3	+	0.8628	NA
nsa-miRN30-5p	GAGAAUAGGAUGAAACUAAACA	22	2	+	1.3719	NA
nsa-miRN31-5p	GUCUCUGGAUUGUAAGGGUU	20	1	−	0.7812	NA
nsa-miRN32-3p	GUAAAUGUUUUGGGACUUCAAGC	23	2	+	0.8763	NA
nsa-miRN33-3p	CCGUUGUUGAUGCAGCGUGG	20	2	+	0.7292	NA
nsa-miRN34-5p	GCGGCGAAGAUGGAAGUAGAG	21	3	+	0.8914	beta-1,3-galactosyltransferase GALT1

**Table 3:** Target enzymes of conserved miRNAs of *N. sativa* in several biosynthesis pathways.

Biosynthetic Pathway	miRNAs	E.C.	Target Enzyme
Carotenoid biosynthesis	nsa-miR167c nsa-miR167b	2.5.1.32	15-cis-phytoene synthase
	nsa-miR157d-3p	1.14.15.24 (LUT5)	beta-carotene 3-hydroxylase
Diterpenoid biosynthesis	nsa-miR394b-5p	1.14.11.12	gibberellin-44 dioxygenase
	nsa-miR159	1.14.11.13	gibberellin 2beta-dioxygenase
	nsa-miR535d	1.14.11.15	gibberellin 3beta-dioxygenase
	nsa-miR169d	1.14.14.58	trimethyltridecatetraene synthase
Phenylpropanoid biosynthesis	nsa-miR8175 nsa-miR156r nsa-miR156f nsa-miR156w nsa-miR156b nsa-miR156p nsa-miR156l	3.1.1	carboxylic ester hydrolases
	nsa-miR164b-5p	1.2.1.68	coniferyl aldehyde dehydrogenase
	nsa-miR156j nsa-miR156e nsa-miR156q nsa-miR164c nsa-miR164c-5p nsa-miR164b-5p	1.11.1.7	peroxidase
Biosynthesis of various plant secondary metabolites	nsa-miR399i	1.1.1.285	3″-deamino-3″-oxonicotianamine
	nsa-miR167c nsa-miR167b	2.4.1.128	scopoletin glucosyltransferase
Terpenoid backbone biosynthesis	nsa-miR156w	2.3.1.9	acetyl-CoA C-acetyltransferase
	nsa-miR535d	2.2.1.7	1-deoxy-D-xylulose-5-phosphate synthase
	nsa-miR157d-3p	2.7.7.60	2-C-methyl-D-erythritol 4-phosphate cytidylyltransferase
	nsa-miR156e	1.17.7.1	(E)-4-hydroxy-3-methylbut-2-enyl-diphosphate synthase (ferredoxin)
		1.17.7.3	(E)-4-hydroxy-3-methylbut-2-enyl-diphosphate synthase (flavodoxin)
	nsa-miR171a-3p	4.2.3.27	isoprene synthase
	nsa-miR156j	3.4. (FACE2)	CAAX prenyl protease 2

**Table 4:** Target enzymes of novel miRNAs of *N. sativa* in several biosynthesis pathways.

Biosynthetic Pathway	miRNA	E.C.	Target Enzyme
Flavonoid biosynthesis	nsa-miRN1-3p nsa-miRN24-3p	2.3.1.133	shikimate O-hydroxycinnamoyltransferase
Phenylpropanoid biosynthesis			
	nsa-mRN10-5p nsa-mRN24-3p	1.11.1.7	peroxidase
Terpenoid backbone biosynthesis	nsa-mRN1-3p	2.3.1.9	acetyl-CoA C-acetyltransferase
		2.7.7.60	2-C-methyl-D-erythritol 4-phosphate cytidylyltransferase
	nsa-mRN10-5p	2.7.4.26	isopentenyl phosphate kinase
Steroid biosynthesis	nsa-miRN24-3p	2.1.1.41 (SMT1/ERG6)	sterol 24-C-methyltransferase
		1.1.1.418	plant 3beta-hydroxysteroid-4alpha-carboxylate 3-dehydrogenase (decarboxylating)
Ubiquinone and other terpenoid–quinone biosynthesis	nsa-miRN16-5p	2.5.1.115	homogentisate phytyltransferase
		2.5.1.116	homogentisate geranylgeranyltransferase

## Data Availability

Publicly available datasets were analyzed in this study. These data can be found here: https://www.ncbi.nlm.nih.gov/search/all/?term=SRX25016763.

## Supplementary Materials

The following supporting information can be downloaded at: https://www.mdpi.com/article/10.3390/plants13192806/s1, Table S1: Summary of conserved miRNAs in *N. sativa*; Table S2: Sequence of primers of *N. sativa* miRNAs used in this study.
