# Supplementary material for: Identification of Black Cumin (Nigella sativa) MicroRNAs by Next-Generation Sequencing and Their Implications in Secondary Metabolite Biosynthesis
Source: Plants (Basel). 2024 Oct 8;13(19):2806. doi: 10.3390/plants13192806 (PMC11478739; doi:10.3390/plants13192806)
Supplement: Supplementary file 1 [file plants-13-02806-s001.zip › plants-3181991-supplementary.pdf]

Supplementary Table S1. Summary of conserved miRNAs in *N. sativa*.

miRNA Family	Name	Sequence (5′-3′)	Length (nt)	Reference miRNA	No. of Mismatches	Read Counts	E Value	Target protein (Related to secondary metabolite biosynthe-sis)
MIR156	nsa-miR156f	UGACAGAAGAGAGAGAGCACA	22	gma-miR156f	0	649	0,0000003	glycerol-3-phosphate 2-O-acyltransferase 6<br>caffeoylshikimate esterase-like
	nsa-miR157d-3p	GCUCUCUAUGCUUCUGUCAUC	21	aly-miR157d-3p	0	175	0,0000003	beta-1,4-mannosyl-glycoprotein 4-beta-N-acetylglucosaminyltransferase<br>protoporphyrinogen oxidase, mitochondrial<br>2-C-methyl-D-erythritol 4-phosphate cytidylyltransferase, chloroplastic-like
	nsa-miR156b	CUGACAGAAGAUAGAGAGCAC	21	smo-miR156b	0	157	0,0000004	caffeoylshikimate esterase-like<br>4-coumarate--CoA ligase-like 9<br>threonine--tRNA ligase, mitochondrial 1
MIR159	nsa-miR159a	UUUGGAUUGAAGGGAGCUCUA	21	ath-miR159a	0	67408	0,0000004	NA
	nsa-miR319b	UUGGACUGAAGGGAGCUCCCU	21	ath-miR319b	0	704	0,0000004	cytochrome P450 CYP82D47
	nsa-miR159	UUUGGCUUGAAGGGAGCUCUA	21	pde-miR159	1	336	0,00004	gibberellin 2-beta-dioxygenase 2-like<br>cytochrome P450 710A11-like<br>glutamate synthase [NADH], amyloplastic-like

MIR160	nsa-miR160g	UGCCUGGCUCCCUGUAUGCCAUC	23	mes-miR160g	0	229	0,00000002	NA
	nsa-miR160d-3p	GCGUGCGAGGAGCCAUGCAU	21	osa-miR160d-3p	1	12	0,0001	NA
	nsa-miR160e-5p	UGCCUGACUCCCUGUAUGCCG	21	osa-miR160e-5p	1	4	0,00004	NA
MIR162_1	nsa-miR162a	UCGAUAAACCUCUGCAUCC	20	gma-miR162a	0	2	0,000002	NA
MIR162_2	nsa-miR162	UCGAUAAACCUCUGCAUCCGG	21	bdi-miR162	0	266	0,0000003	cytochrome P450 83B1-like
MIR164	nsa-miR164b-5p	UGGAGAAGCAGGGCACGUGCA	21	ath-miR164b-5p	0	2422	0,0000004	UDP-glucuronic acid decarboxylase 2<br>UDP-glycosyltransferase 90A1<br>7-deoxyloganetin glucosyltransferase-like
	nsa-miR164g-3p	CACGUGCUCCCCUUCUCCA	21	zma-miR164g-3p	0	76	0,000002	7-deoxyloganetic acid glucosyltransferase-like
	nsa-miR164c-5p	UGGAGAAGCAGGUCACGUGCG	21	ath-miR164c-5p	1	46	0,00003	7-deoxyloganetin glucosyltransferase<br>UDP-glucuronic acid decarboxylase 2<br>7-deoxyloganetin glucosyltransferase-like
MIR166	nsa-miR166a	UCGGACCAGGCUUCAUUCCCC	21	pta-miR166a	0	7508	0,0000004	E4 SUMO-protein ligase PIAL2-like
	nsa-miR166k	UCUCGGACCAGGCUUCAUUCC	21	gma-miR166k	0	5040	0,0000004	anthocyanidin 3-O-glucosyltransferase 2-like<br>cytochrome P450 704B1

	nsa-miR166c-5p	GGAAUGUUGUCUGGCUCGAGG	21	gma-miR166c-5p	0	380	0,0000003	cysteine-rich receptor-like protein kinase 29 protein HOTHEAD-like phosphatase IMPL1, chloroplastic
MIR167	nsa-miR167h	UUGAAGCUGCCAGCAUGAU	24	gma-miR167h	0	4	0,000005	NA
	nsa-miR167b	UGAAGCUGCCAGCAUGAUCUA	22	bna-miR167b	0	368	0,0000003	aldehyde dehydrogenase family 3 phytoene synthase 2, chloroplastic scopoletin glucosyltransferase
MIR167_1								
	nsa-miR167c	UGAAGCUGCCAGCAUGAUCUC	21	vvi-miR167c	0	18	0,0000002	aldehyde dehydrogenase family 3 member F1-like phytoene synthase 2, chloroplastic-like scopoletin glucosyltransferase-like
	nsa-miR167f-3p	AGAUCAUGUGGCAGUUUCACC	21	ptc-miR167f-3p	0	16	0,0000002	NA
	nsa-miR168b-5p	UCGCUUGGUGCAGGUCGGGAA	21	ath-miR168b-5p	0	2280	0,0000004	7-deoxyloganetin glucosyltransferase
MIR168	nsa-miR168a-3p	CCCGCCUUGCAUCAACUGAAU	21	ath-miR168a-3p	0	7	0,0000002	cytochrome P450 83B1-like
	nsa-miR168c-5p	UCGCUUGGUGCAGGUCGGGAC	21	bra-miR168c-5p	0	1	0,0000001	NA
MIR169_1	nsa-miR169h	UAGCCAAGGAUGACUUGCCUG	21	ath-miR169h	0	2	0,0000002	NA
MIR169_2	nsa-miR169d	GCCAAGGAUGACUUGCCGGU	23	gma-miR169d	0	57	0,0000006	cytochrome P450 CYP82D47-like

	nsa-miR169b-5p	CAGCCAAGGAUGACUUGCCGG	21	ath-miR169b-5p	0	21	0,0000001	oleoyl-acyl carrier protein thioesterase, chloroplastic
	nsa-miR169r-3p	GCAAGUUGUCCUUGGCUACA	21	zma-miR169r-3p	0	14	0,0000006	NA
	nsa-miR171d	UGAUUGAGCCGUGCCAAUAUC	21	cpa-miR171d	0	7934	0,0000004	NA
MIR171_1	nsa-miR171q	UUGAGCCGUGCCAAUAUCACA	21	gma-miR171q	0	1925	0,0000004	O-acyltransferase WSD1-like
	nsa-miR171a-3p	UGAUUGAGCCGCGCCAAUAUC	21	ath-miR171a-3p	0	477	0,0000003	probable terpene synthase 12
MIR171_2	nsa-miR171b-3p	CGAGCCGAAUCAAUAUCACUC	21	gma-miR171b-3p	0	2	0,0000002	O-acyltransferase WSD1-like
MIR172	nsa-miR172a	AGAAUCUUGAUGAUGCUGCAU	21	ath-miR172a	0	22	0,0000001	NA
	nsa-miR390b	AAGCUCAGGAGGGAUAGCGCC	21	ppt-miR390b	0	643	0,0000002	NA
MIR390	nsa-miR390d-3p	CGCUAUCCAUCCUGAGUUUUA	21	ptc-miR390d-3p	0	11	0,0000002	NA
	nsa-miR390c-5p	AGCUCAGGAGAGAUAGCGCC	21	ppt-miR390c-5p	1	1	0,0001	NA
	nsa-miR393	UCCAAAGGGAUCGCAUUGAUCU	22	ghr-miR393	0	43	0,00000004	NA
MIR393	nsa-miR393a	AUCCAAAGGGAUCGCAUUG	21	ppe-miR393a	0	2	0,000002	NA
	nsa-miR393c-3p	AUCAUGCUAUCCCUUUGGAUU	21	gma-miR393c-3p	0	1	0,0000002	NA
MIR394	nsa-miR394b-5p	UUGGCAUUCUGUCCACCUCC	20	ath-miR394b-5p	0	55	0,0000009	UDP-glycosyltransferase 73C6-like<br>serine--tRNA ligase<br>gibberellin 20 oxidase 1-D
MIR395	nsa-miR395a	CUGAAGGGUUUGGAGGAACUC	21	ptc-miR395a	0	17	0,0000002	NA
	nsa-miR395i	UGAAGUGUUUGGGGGAACUC	21	gma-miR395i	0	1	0,0000006	NA
MIR396	nsa-miR396	UUCCACAGCUUUCUUGAACUU	21	pta-miR396	0	6521	0,0000004	UDP-glycosyltransferase 86A2

	nsa-miR396b-3p	GUUCAAUAAAGCUGUGGGAAA	21	zma-miR396b-3p	0	271	0,0000003	NA
	nsa-miR396a-5p	UUCCACAGCUUUCUUGAACUG	21	ath-miR396a-5p	0	63	0,0000004	UDP-glycosyltransferase 86A2
	nsa-miR398c	UGUGUUCUCAGGUCGCCCCUG	21	gma-miR398c	0	9	0,0000002	NA
MIR398	nsa-miR398a-5p	GGAGUGACACUGAGAACACAAG	22	mtr-miR398a-5p	0	3	0,00000004	NA
	nsa-miR398c-3p	GUGUUCUCAGGUCACCCCUG	21	ath-miR398c-3p	0	2	0,0000006	NA
MIR399	nsa-miR399i	CGCCAAAGGAGAGUUGCCCUG	21	vvi-miR399i	0	11	0,0000002	dol-P-Man:Man(5)GlcNAc(2)-PP-Dol alpha-1,3-mannosyltransferase non-functional NADPH-dependent codeinone reductase 2-like
	nsa-miR399c-3p	CAAAGGAGAGUUGCCCUG	21	ath-miR399c-3p	0	1	0,000009	NA
MIR414	nsa-miR414	GACGAAGAUGAUGAAGAUG	21	ath-miR414	1	1	0,0005	NA
MIR477	nsa-miR477f	CUCUCCUUCAAAGGCUUC	20	aqc-miR477f	0	3	0,000009	glutamate dehydrogenase B 7-deoxyloganetin glucosyltransferase-like
	nsa-miR482-5p	GGAAUGGGCGGAUUGGGAAGC	22	pvu-miR482-5p	1	15	0,00004	glutamate dehydrogenase B 7-deoxyloganetin glucosyltransferase-like
MIR482	nsa-miR2118	UUGCCGAUUCCACCCAUUCCUA	22	vun-miR2118	0	8	0,00000004	NA
	nsa-miR482a-3p	UUCCCAAUUCUGCCCAUUCCU	24	gma-miR482a-3p	1	3	0,00004	NA
MIR530	nsa-miR530b	UGCAUUUGCACCUGCAUC	20	ptc-miR530b	0	3	0,000009	NA

	nsa-miR535d	UGACAACGAGAGAGAGCACGC	21	ppt-miR535d	0	4090	0,0000003	NA
MIR535	nsa-miR535	UGACAACGAGAGAGAGCACGCG	22	aqc-miR535	0	22	0,00000009	NA
	nsa-miR535d	UGACCACGAGAGAGAGCACGC	21	mdm-miR535d	1	20	0,00004	NA
MIR818	nsa-miR1130b-3p	UCUUAUAUUAUAGGACGGAGG	21	tae-miR1130b-3p	1	2	0,00004	NA
MIR827	nsa-miR827	UUAGAUGAUCAUCAACAAACA	21	nta-miR827	1	40	0,00004	NA
MIR827_5	nsa-miR827-5p	UUUGUUGAUGGUCAUCUA	21	stu-miR827-5p	0	6	0,00001	NA
MIR828	nsa-miR828a	UCUUGCUCAAAUGAGUAUUCCA	22	gma-miR828a	0	1	0,00000006	NA
MIR835	nsa-miR835-5p	UUCUUGCAUAUGUUCUUU	21	ath-miR835-5p	0	1	0,00002	NA
MIR838	nsa-miR838-3p	GUGCAAGAAGAAGAAGAA	21	aly-miR838-3p	0	1	0,000009	NA
MIR845_3	nsa-miR845b	AUCAAUUGGUAUCAGAGC	21	vvi-miR845b	0	1	0,00001	NA
MIR1507	nsa-miR1507a	UCUCAUUCCAUACAUCGUC	22	gma-miR1507a	0	2	0,000002	NA
MIR1515	nsa-miR1515a	UCAUUUUGCGUGCAAUGAUCUG	22	gma-miR1515a	0	3	0,00000004	NA
MIR1863	nsa-miR1863b	UUAACAUGGUAUCAGAGCU	24	osa-miR1863b	0	1	0,000003	NA
MIR2111	nsa-miR2111c	UAAUCUGCAUCCUGGGGUUU	21	bna-miR2111c	0	1	0,0000006	NA
MIR2673	nsa-miR2673b	GAAGAGGAAGAGGAAGAGG	22	mtr-miR2673b	0	1	0,000003	NA
MIR2950	nsa-miR2950-5p	UUCCAUCUCUUGCACACUGGA	21	vvi-miR2950-5p	0	81	0,0000003	delta(14)-sterol reductase 3-epi-6-deoxocathasterone 23-monooxygenase
MIR3630	nsa-miR3630-3p	UUGGGAAUCUCUCUGAUGCA	22	vvi-miR3630-3p	0	26	0,000001	NA
MIR5067	nsa-miR5049c	UCCGUCCCAAAAUGAUUGUCU	23	hvu-miR5049c	1	2	0,00004	NA
MIR5225	nsa-miR5225a	GUCGCAAGAGAGAUGACAC	22	mtr-miR5225a	1	2	0,0006	NA
MIR6476	nsa-miR6476a	UCAGUGGAGAUGGAACAUGA	20	ptc-miR6476a	1	23	0,0003	NA
MIR7494	nsa-miR7494b	AGAGAGAGAAGCAGAAGAGAA	23	gra-miR7494b	1	1	0,00008	NA

MIR7504	nsa-miR7504d	GAAAUAAAAUCUGAUUUGUCA	24	gra-miR7504d	1	1	0,00004	NA
MIR8005	nsa-miR8005c	AAACUUUAAACCUUAAACU	24	stu-miR8005c	1	1	0,001	NA
MIR8762	nsa-miR8762d	UUGUUGACUUUGAUGAUGUG	24	gra-miR8762d	1	1	0,0001	NA
	nsa-miR6300	GUCGUUGUAGUAUAGUGG	18	gma-miR6300	0	81625	0,00002	NA
NA	nsa-miR6478	CCGACCUUAGCUCAGUUGGU	21	ptc-miR6478	0	1127	0,000001	UDP-glycosyltransferase 74E2-like
	nsa-miR894	CGUUUCACGUCGGGUUCACC	20	ppt-miR894	0	1102	0,000001	NA

Supplementary Table S2: Sequence of primers of *N. sativa* miRNAs used in this study.

**Novel miRNA**	**Specific primer sequence**
nsa-miRN1	5´TCTCTCTTCCTGATCCTCCT3’
nsa-miRN29	5´GAAGTTCTCGGGGGTGTTTGA3’
nsa-miRN32	5´GTAAATGTTTTGGGACTTCAAGC3’
nsa-miRN34	5´GCGGCGAAGATGGAAGTAGAG3’
**Conserved miRNA**	**Specific primer sequence**
nsa-miR164d	5´TGGAGAAGCAGGGCACGTGCA3’
nsa-miR166a	5´TCGGACCAGGCTTCATTCCCC3’
nsa-miR167b	5´TGAAGCTGCCAGCATGATCTA3’
nsa-miR171a	5´TGATTGAGCCGCGCCAATATC3’
nsa-miR390b	5´AAGCTCAGGAGGGATAGCGCC3’
nsa-miR396	5´TTCCACAGCTTTCTTGAACTT3’
nsa-miR159a	5´TTTGGATTGAAGGGAGCTCTA3’
